# Renoprotective effects of Gushen Jiedu capsule on diabetic nephropathy in rats

**DOI:** 10.1038/s41598-020-58781-2

**Published:** 2020-02-06

**Authors:** Lei Zhang, Zhirui Yang, Yidan Zhao, Xinyu Yang, Xintong Meng, Juan Liu, Yi Liu, Can Yan, Dan Yan

**Affiliations:** 1 Beijing Shijitan Hospital, Capital Medical University, Beijing, 100038 China; 2 Beijing Key Laboratory of Bio-characteristic Profiling for Evaluation of Rational Drug Use, Beijing, 100038 China; 3 Guangzhou University of Chinese Medicine, School of Basic Medical Sciences, Guangzhou, 510006 China

**Keywords:** Chronic kidney disease, Kidney diseases

## Abstract

Gushen Jiedu capsule (GSJD) is a formula that has been widely used in traditional Chinese medicine for the prevention and treatment of diabetic nephropathy (DN). However, the mechanism underlying the protective effects of GSJD on DN is still unclear. This study was performed to clarify the therapeutic effects of GSJD on DN and its underlying mechanisms. High-fat diet- and streptozotocin-induced DN rats were treated with or without GSJD suspension by gavage for 8 weeks, and biochemical changes in blood and urine were analysed. Kidneys were isolated for histological, TUNEL and Western blot analysis. Compared to the DN group, the GSJD-treated groups exhibited decreased urinary albumin, ameliorated renal dysfunction, including serum creatinine and blood urea nitrogen, and attenuated total cholesterol, triglyceride and total protein levels. However, there were no significant effects of GSJD on body weight, fasting blood glucose or albuminuria. Histology showed that GSJD could retard the progression of DN and decrease the apoptosis rate from 52% to less than 20%. Western blot analysis showed that GSJD could regulate the mitochondrial apoptotic pathway by downregulating the expression of Bax and upregulating the expression of BCL-2 in the kidneys of DN rats. Moreover, the Akt pathway, an upstream signalling pathway of the BCL-2 family, was also ameliorated by GSJD. Further, the podocyte foot process markers podocin and nephrin were upregulated by GSJD in DN rats. This study demonstrated that GSJD might play a renoprotective role by inhibiting apoptosis and regulating the mitochondrial apoptotic and Akt pathways during pathological changes in DN.

## Introduction

Diabetic nephropathy (DN) is a diabetes-induced microvascular complication that is responsible for 50% of all cases of end-stage renal disease (ESRD) and is characterized by hyperglycaemia, continuous albuminuria, high blood pressure and impaired renal function^1–3^. Additionally, DN is a major risk factor for mortality in patients with diabetic complications worldwide^4,5^. Hence, the prevention and treatment of DN have become a serious health issue, and the development of DN medications would be beneficial.

Traditional Chinese medicine (TCM) has been used in the treatment of diabetes and related complications for thousands of years in China, which emphasizes the whole regulatory action on the human body^6,7^. Thus, TCM formulae have become an important source for the discovery of new medications to prevent and treat DN^8,9^. For instance, the NaoXinTong capsule^10^ improves glomerular function, inhibits extracellular matrix accumulation, activates the insulin signalling pathway and improves glucose metabolism in *db/db* mice, suggesting a favourable effect on kidney function.

The Gushen Jiedu capsule (GSJD) originated from the classic kidney-tonifying prescription of “Shuilu Erxian Dan” in the Song Dynasty (1170 AD) in China (Supplementary Fig. S1), containing Semen *Euryales*, Fructus *Rosa laevigata*, Rhizome *Coptis chinensis*, Rhizome *Rheum tanguticum*, Radix *Astragalus membranaceus* and Radix *Angelica sinensis*. In the formula, Semen *Euryales* and Fructus *Rosa laevigata* were the monarch components and Rhizome *Coptis chinensis* and Rhizome *Rheum tanguticum* were the minister components, while Radix *Astragalus membranaceus* and Radix *Angelica sinensis* were the adjuvant components. GSJD has been used to treat early-stage DN in the clinic for over twenty years in China and was developed into a hospital preparation in recent years. Additionally, it was mainly suitable for kidney yang deficiency patients^11^. Notably, our results have demonstrated that GSJD could significantly decrease the levels of serum creatinine (Scr), blood urea nitrogen (BUN) and albuminuria and retard renal pathological changes. However, the renoprotective mechanism of GSJD is still unclear and must be scientifically explained.

A host of studies have indicated that apoptosis is the ultimate consequence of a number of biological processes that lead to DN^5,12^. BCL-2 family proteins are primary regulators of the mitochondrial apoptotic pathway and determine cellular survival or death decisions by regulating the integrity of the mitochondrial outer membrane^13^. Protein kinase B (Akt), the upstream signalling pathway of the mitochondrial apoptotic pathway, has been demonstrated to regulate protein synthesis and cellular growth^14,15^. Therefore, the activation of the mitochondrial apoptotic pathway and Akt pathway is thought to play a key role in the apoptotic pathway^16,17^. In our present study, we demonstrated that GSJD could remarkably reduce apoptosis in renal tissue cells. Therefore, the pronounced renoprotective effect of GSJD against DN prompted us to test whether GSJD attenuates DN by inhibiting apoptosis and regulating the expression of the BCL-2 family and Akt.

Hence, in this study, we attempted to investigate the effects of GSJD, with a focus on its renoprotective potential and underlying anti-apoptotic mechanisms, by using a high-fat diet (HFD)- and streptozotocin (STZ)-induced animal model.

## Results

### GSJD improved the general condition and biochemical parameters of DN rats

**Figure 1:**
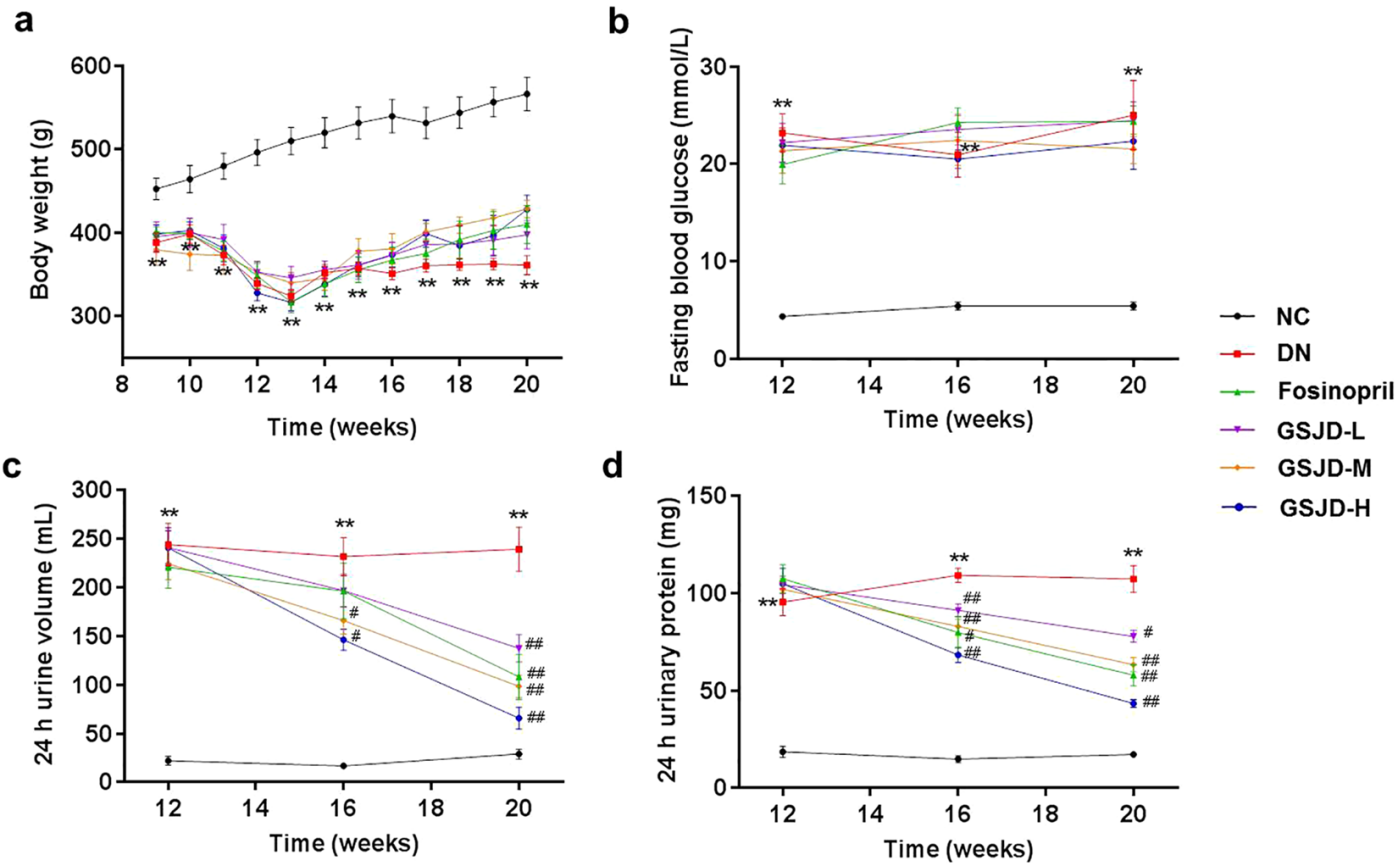
Effects of GSJD on general parameters in rats after injection of streptozotocin. (**a**) Body weight, (**b**) fasting blood glucose, (**c**) 24 h urine volume and (**d**) 24 h urinary protein. Data were analysed by *t*-tests (**a** and **b**) and one-way ANOVA (**c** and **d**) and presented as the mean ± SEM (*n* = 6). **P < 0.01 DN *vs*. NC, ^#^*P* < 0.05 *vs*^.^ DN, and ^##^*P* < 0.01 *vs*. DN.

By the end of 8 weeks of HFD feeding, the average body weight of the model group (high-fat diet-fed group) was similar to that of the normal group (Supplementary Fig. S2). After the DN model was established successfully, rats in the normal control group (NC group) exhibited progressive body weight gain, while rats in the DN group showed less body weight gain (Fig. 1a). Nevertheless, treatments with either fosinopril or GSJD did not prevent the reduction in body weight. In addition, the fasting blood glucose (FBG) level of the DN group was significantly higher than that of the NC group, and the treatments with either fosinopril or GSJD did not reverse the elevation of FBG in these groups (Fig. 1b). Fortunately, fosinopril, a medium dose of GSJD, and a high dose of GSJD reduced the 24 h urinary protein level of rats (*P* < 0.01) (Fig. 1c,d), while the rats in the DN group had a significantly elevated 24 h urinary protein level (*P* < 0.01).

**Figure 2:**
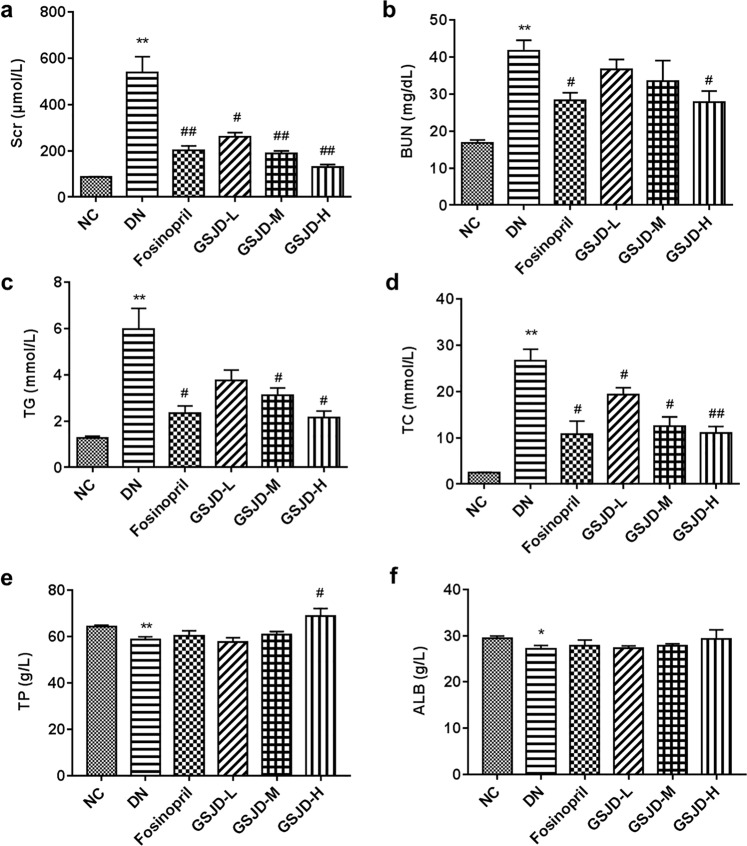
Effects of GSJD on biochemical parameters in rats. (**a**) Scr, (**b**) BUN, (**c**) TG, (**d**) TC, (**e**) TP and (**f**) ALB. Data were analysed by one-way ANOVA and presented as the mean ± SEM (*n* = 6). *P < 0.05 DN *vs*. NC, **P < 0.01 DN *vs*. NC, ^#^*P* < 0.05 *vs*. DN, and ^##^*P* < 0.01 *vs*. DN.

Previous reports have shown that STZ-induced HFD-fed rats produced high levels of Scr, BUN and blood lipids and low levels of total protein (TP) and serum albumin (ALB)^18^. We measured the relative biochemical parameters within the serum of each group after GSJD treatment for 8 weeks. As shown in Fig. 2, the Scr, BUN, triglyceride (TG), total cholesterol (TC), TP and ALB levels of the DN group remained at normal levels throughout the experiment. After successful modelling, the Scr, BUN, TG and TC levels in the DN group were notably higher than those in the NC group, while TP and ALB levels decreased slightly. After GSJD treatment for 8 weeks, the group treated with a high dose of GSJD showed markedly ameliorated Scr, BUN, TG, TC and TP levels compared to the DN group (*P* < 0.05) (Fig. 2a–e). There were no significant differences in ALB levels among the fosinopril and all GSJD treatment groups (Fig. 2f).

### GSJD alleviated renal histopathology and ultrastructural changes in DN rats

**Figure 3:**
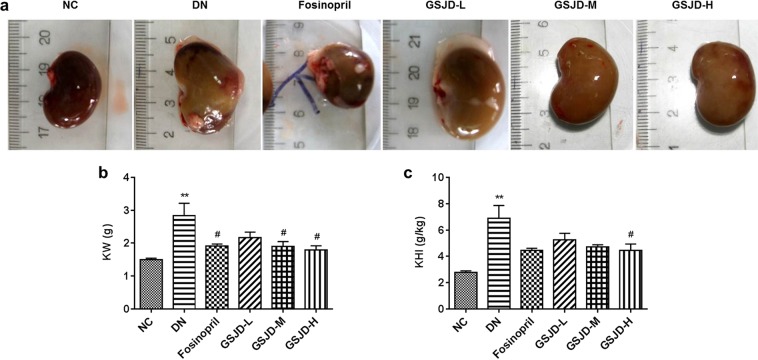
Effects of GSJD on (**a**) renal shape, (**b**) KW and (**c**) KHI in rats. Data were analysed by one-way ANOVA and presented as the mean ± SEM (*n* = 6). **P < 0.01 DN *vs*. NC and ^#^*P* < 0.05 *vs*. DN.

At the end of the study, the kidneys in the DN group markedly swelled and weighed more than those in the NC group (Fig. 3a). After GSJD treatment for 8 weeks, the kidney enlargement, kidney weight (KW) and kidney hypertrophy index (KHI) of the GSJD high-dosage (GSJD-H) group rats were obviously improved, and the differences in these parameters between the GSJD-H and DN groups were statistically significant (*P* < 0.05). In addition, kidney enlargement was partly ameliorated in the GSJD medium dosage (GSJD-M) group rats and fosinopril group rats. However, there were no significant improvements in KHI by fosinopril or low-dose or medium-dose GSJD treatment (Fig. 3a–c).

**Figure 4:**
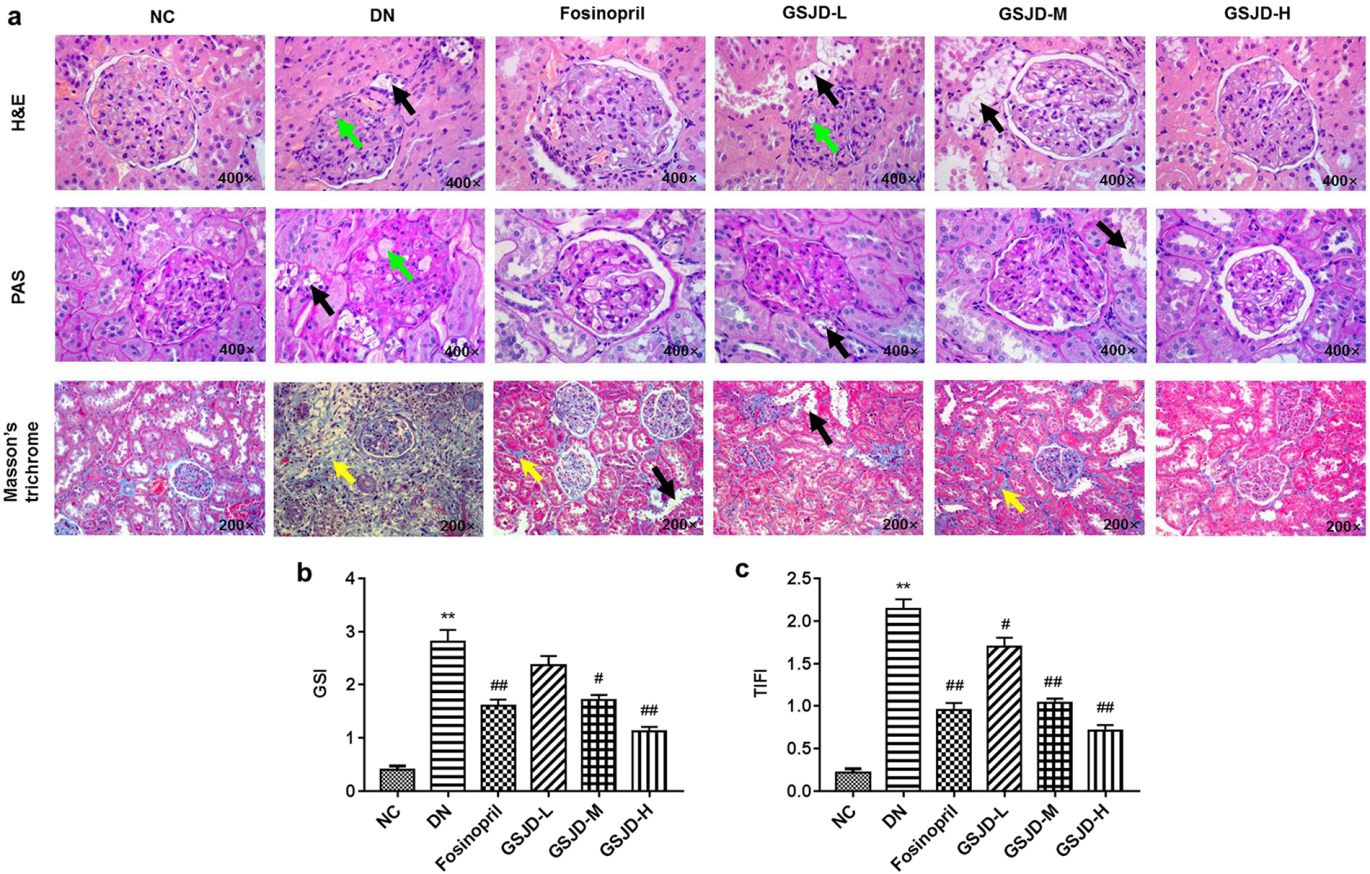
Effects of GSJD on renal pathology of DN rats. (**a**) Representative microstructural images of different groups. (H&E and PAS staining, original magnification 400×; Masson’s trichrome staining, original magnification 200×; black arrow: tubular vacuolar degeneration, green arrow: glomerular hyaline degeneration, yellow arrow: tubulointerstitial fibrosis); (**b**) GSI; (**c**) TIFI. Data were analysed by one-way ANOVA and presented as the mean ± SEM (*n* = 6). **P < 0.01 DN *vs*. NC, ^#^*P* < 0.05 *vs*^.^ DN, and ^##^*P* < 0.01 *vs*. DN.

Since kidney structural abnormalities are the major cause of renal dysfunction^1,4,19^, we examined the renal microstructure with haematoxylin-eosin (H&E), periodic acid-Schiff (PAS) and Masson’s staining. As shown in Fig. 4a, DN rats displayed obvious mesangial matrix expansion, thickened glomerular basement membranes (GBM), severe glomerulosclerosis, glomerular hyaline degeneration, tubular vacuolar degeneration and tubulointerstitial fibrosis compared to NC rats. Moreover, there was significant neutrophil and lymphocytic infiltration and fibrosis in the tubulointerstitium in DN rats. Compared with the DN group, the group treated with low-dose GSJD (GSJD-L) did not exhibit any noticeable effect on the renal histopathological changes. Glomerular pathology was partially repaired by GSJD-M treatment, but tubular vacuolar degeneration was still observed. Further, both GSJD-H and fosinopril treatment markedly attenuated the abnormalities in both the glomerular and tubular structures in rats. Semiquantitative data further confirmed these observations (Fig. 4b,c).

**Figure 5:**
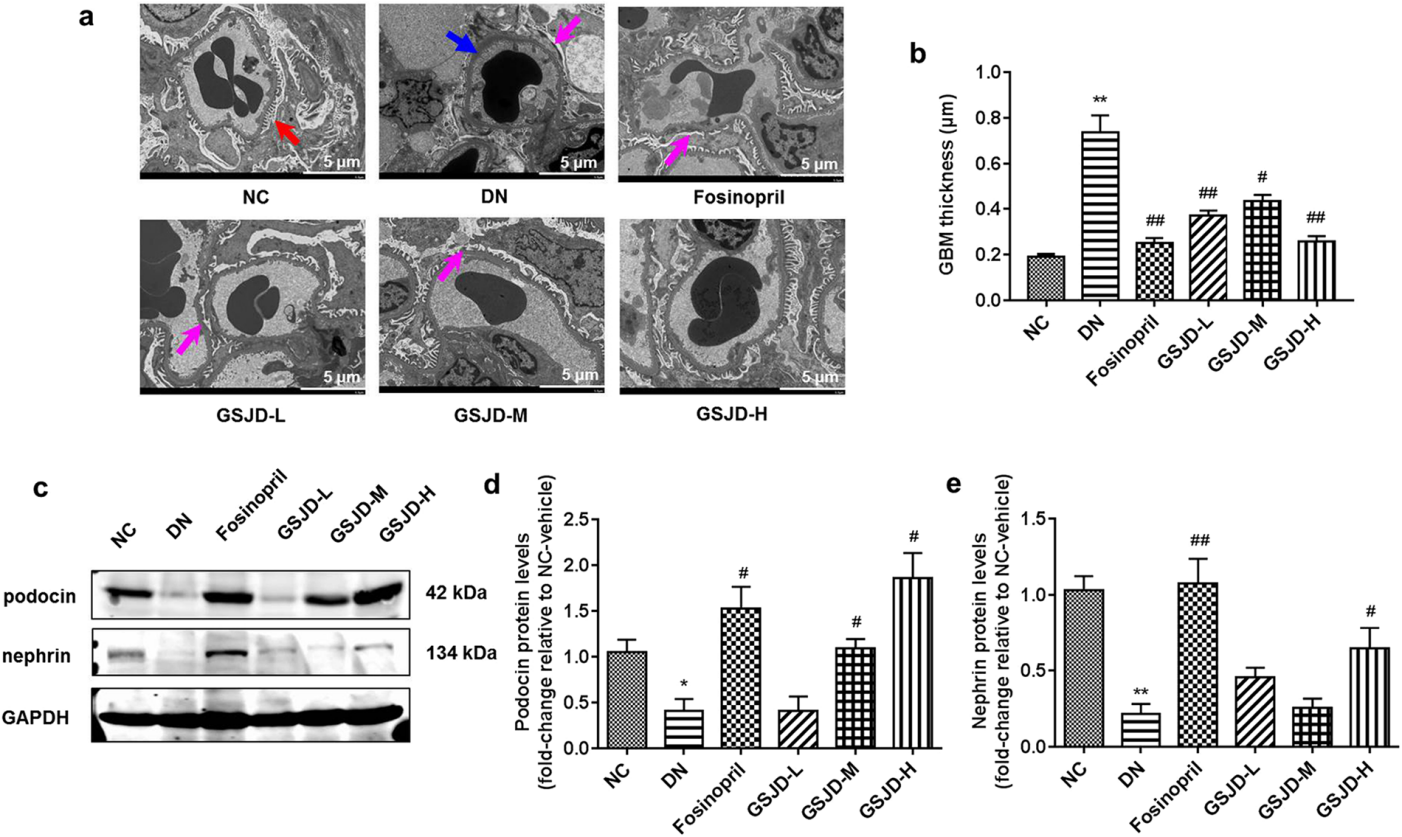
Effects of GSJD on GBM thickness, foot process form and podocin and nephrin protein expression in the kidneys of rats. (**a**) Representative ultrastructural changes in different groups (Scale bars 5 μm; red arrow: normal foot process, blue arrow: thickened GBM and effacement of foot process, pink arrow: fusion of foot process); (**b**) Quantification of GBM thickness; (**c**) Characteristics of podocin and nephrin protein expression in each group; (**d**,**e**) Quantitative analysis of podocin and nephrin protein expression in each group. Data were analysed by one-way ANOVA and presented as the mean ± SEM (*n* = 3). *P < 0.05 DN *vs*. NC, **P < 0.01 DN *vs*. NC, ^#^*P* < 0.05 *vs*. DN, and ^##^*P* < 0.01 *vs*. DN.

To further examine the actions of GSJD on GBM thickness, the structure of podocytes, expression of podocyte proteins in renal tissues, and ultrastructural changes were observed by transmission electron microscopy (TEM), and the expression levels of podocin and nephrin were analysed by Western blot analysis. As shown in Fig. 5a, the NC group was characterized by glomerular structural integrity and clear podocyte morphology. The GBM and mesangial matrix had smooth and flat surfaces, and the podocytes and GBM were in close contact. In contrast, the DN group displayed thickening of the GBM (*P* < 0.01) (Fig. 5b), proliferation of mesangial cells and matrix, fusion or effacement of foot processes and disordered arrangement of podocytes relative to the NC group. In the GSJD-M group, foot process fusion was reduced, and the GBM, mesangial cells and matrix showed fewer pathological changes. Notably, the GSJD-H and fosinopril groups showed significant improvement of renal pathological changes compared with the DN group. However, foot process fusion and a thickened GBM were still observed in the GSJD-L group. In addition, podocin and nephrin were downregulated in the DN group (*P* < 0.05), indicating a damaged glomerular filtration barrier. Nevertheless, GSJD restored the expression of podocin and nephrin (Fig. 5c–e). Somewhat unexpectedly, the alteration of nephrin was not dose dependent.

### GSJD decreased apoptosis in DN rats

**Figure 6:**
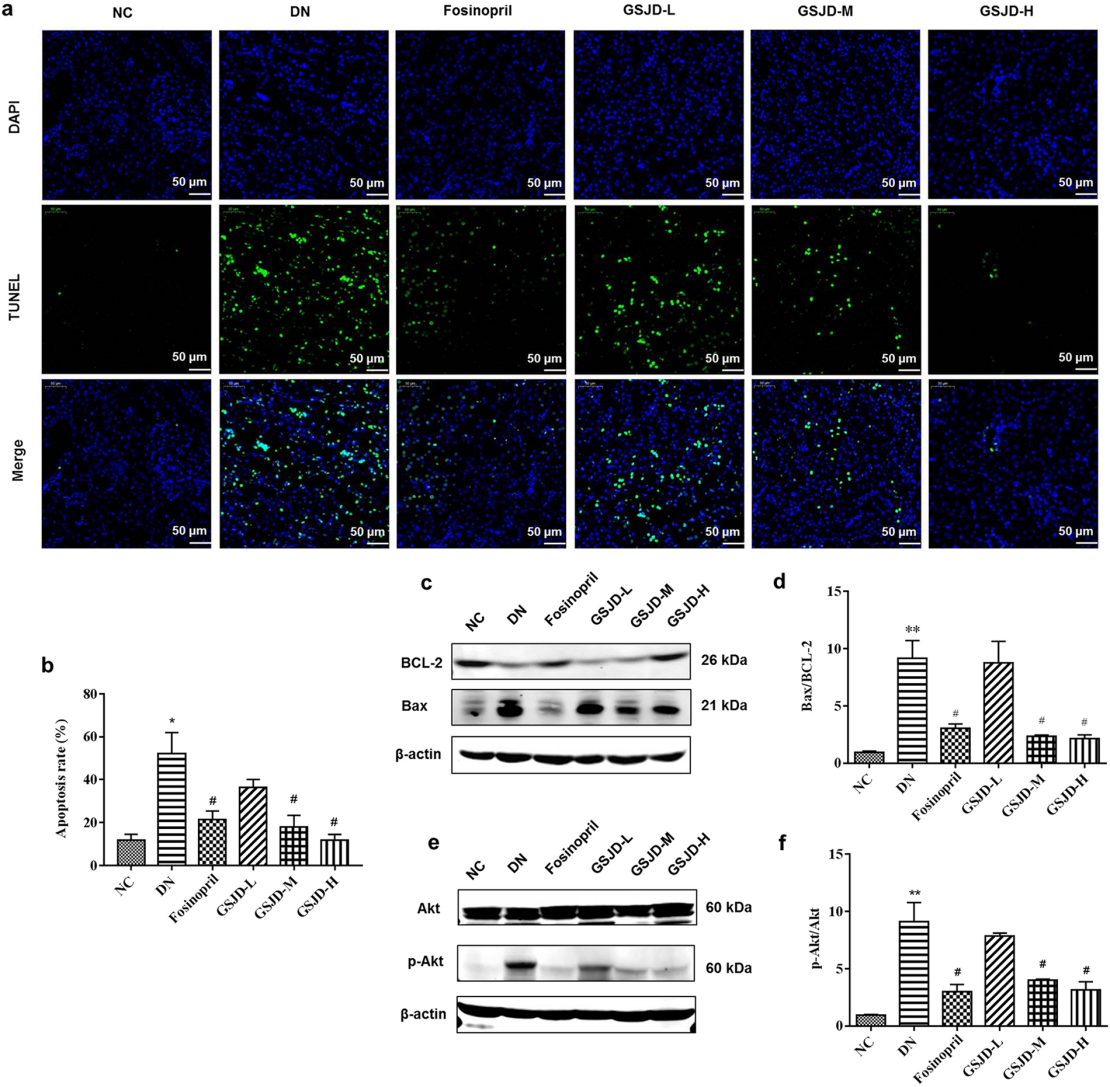
GSJD ameliorated apoptosis in the kidneys of rats. (**a**) Representative images of TUNEL staining among 6 groups (scale bars 50 μm); (**b**) Quantification of the apoptosis rate in each group; (**c**-**f**) Western blot analysis of BCL-2, Bax, Akt and p-Akt protein expression in each group, with quantification. Data were analysed by one-way ANOVA and presented as the mean ± SEM (*n* = 3). *P < 0.05 DN *vs*. NC, **P < 0.01 DN *vs*. NC and ^#^*P* < 0.05 *vs*. DN.

Apoptosis participates in the pathogenesis of DN and is an adaptive response in cells under environmental stress^20^, which is now clearly recognized as a major cause of podocyte loss and albuminuria in DN^21^. The mitochondrial apoptotic and Akt pathways have been demonstrated to be major functional pathways in apoptosis^13,22,23^. To investigate the role of apoptosis, the alteration of apoptosis in DN was analysed by TUNEL staining and Western blot. Interestingly, the number of apoptotic cells (observed by green fluorescence) within the glomeruli and renal tubules was significantly increased in DN rats compared with NC rats, which was dose-dependently rescued by GSJD (Fig. 6a). However, there was no statistically significant difference in the apoptosis rate between the GSJD-L group and DN group (Fig. 6b). Notably, we found that the expression of Bax was upregulated and the expression of BCL-2 was markedly decreased in the DN group, whereas this change was prevented by GSJD treatment (*P* < 0.05) (Fig. 6c,d). In addition, the expression of p-Akt was remarkably increased in kidney tissues in the DN group. Our results showed a significant reduction in p-Akt in the GSJD-H treatment group (*P* < 0.05) (Fig. 6e,f). Unfortunately, however, the alteration of Bax was not dose dependent. Altogether, these data indicated that the mitochondrial apoptotic pathway and Akt pathway were activated in DN rats. GSJD treatment could alter the excessive apoptosis of renal tissues and rescue renal function.

## Discussion

In this study, we used HFD- and STZ-induced DN rats to investigate the pharmacodynamic actions of GSJD on DN progression and explored the mechanisms underlying the beneficial actions of GSJD *in vivo*. Clearly, our results demonstrated that GSJD exerted an excellent renoprotective effect on DN development, as evidenced by enhanced renal function and ameliorated renal pathological changes. In addition, the expression of podocyte proteins, which are considered markers of renal injury in numerous studies^24,25^, was markedly decreased in DN rats. Notably, following GSJD treatment, the expression of p-Akt and Bax was significantly decreased, while the expression of BCL-2 was markedly increased, concomitant with a notable attenuation of the apoptosis rate. Here, our results suggest that the beneficial effect of GSJD on DN is associated with apoptosis inhibition through the mitochondrial apoptotic and Akt pathways.

DN is a diabetic complication that develops in approximately one-third of all patients with type 1 and half of those with type 2 diabetes and is characterized by elevated urinary albumin, impaired renal function, and disordered glucose-lipid metabolism^1^. Urinary albumin is the earliest indicator and a major risk factor for progressive renal dysfunction in DN. In this study, HFD- and STZ-induced DN rats displayed increases in urinary albumin levels, elevated Scr, BUN, TG and TC levels, decreased TP and ALB levels, kidney enlargement, mesangial matrix expansion, GBM thickening, glomerulosclerosis, tubulointerstitial fibrosis and fusion or effacement of foot processes, indicating impaired renal function, disordered lipid metabolism and progression of DN in our animal model. Our data clearly indicated that GSJD treatment obviously attenuated albuminuria, reduced Scr, BUN, TG and TC levels, evaluated TP levels, and ameliorated kidney enlargement, KHI and histopathology, suggesting the profound renoprotective effects of GSJD on DN. Nevertheless, GSJD treatment had no obvious effects on body weight or FBG, which was consistent with the investigation of astragaloside IV^26^ and Chaihuang-Yishen granule^27^ in the attenuation of DN. Therefore, we believe that the repaired renal function of GSJD may be a direct effect on renal tissues.

To our knowledge, podocytes are particularly imperative for sustaining glomerular filtration barrier function, and podocyte injury plays a vital role in the development of albuminuria in DN^12^. Podocin and nephrin are important elements of the slit diaphragm in podocytes, as they not only are the basis of an important intracellular signalling hub but also are directly involved in podocyte survival pathways. Hyperglycaemia-induced endocytosis results in the loss of podocin and nephrin in DN rats, leading to podocyte injury and proteinuria^1,28,29^. In our study, we observed reduced expression of podocin and nephrin, as well as foot process fusion and effacement, indicating podocyte dysfunction in DN rats. Further study revealed that GSJD clearly regulated the expression of these proteins in GSJD-treated rats compared with DN rats. Hence, our results suggest that the protective effect of GSJD may be targeting podocytes.

**Figure 7:**
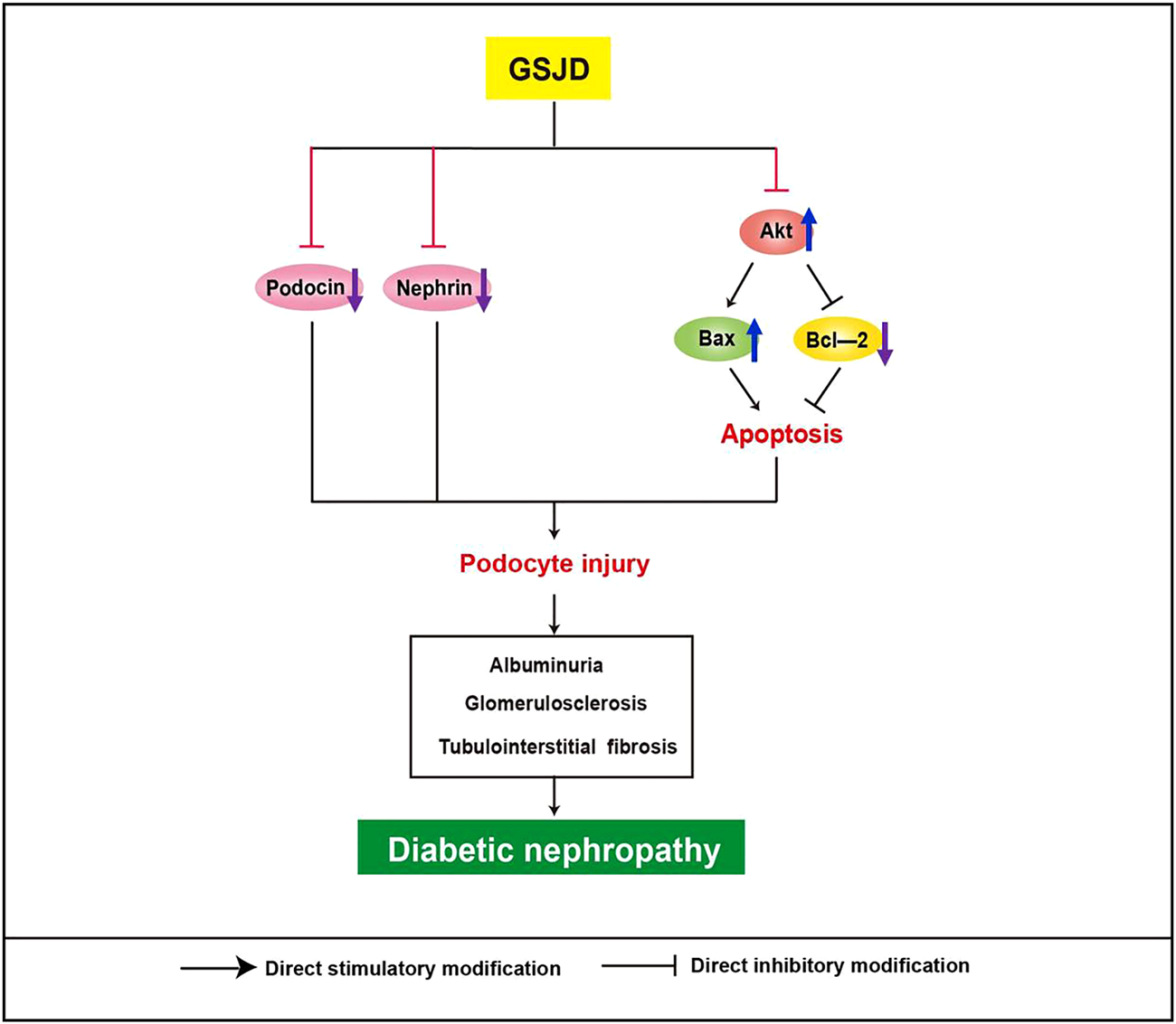
The possible mechanism of GSJD in attenuating DN.

Apoptosis is an important factor during the process of DN, and it participates in podocyte injury and can be initialized through the intrinsic pathway (mitochondrial apoptotic pathway)^30,31^. Emerging evidence has revealed the importance of the BCL-2 family in promoting mitochondrial apoptosis^13,32^. Our data indicated that the expression of BCL-2 was markedly downregulated, while the expression of Bax was significantly increased, in the DN group and both were distinctly restored after GSJD treatment for 8 weeks. Interestingly, similar to our finding, Sohn *et al*.^33^ and Kaeidi *et al*.^34^ reported that treatment with *Aster koraiensis* and *Satureja khuzestanica* downregulated the expression of Bax and upregulated the expression of BCL-2 in diabetic rats. Akt, a serine/threonine-specific protein kinase, plays a key role in the signalling pathways regulating apoptosis and cell growth by activating a series of downstream signalling pathways. Growing evidence suggests that the activity or expression of Akt is related to the regulation of BCL-2 family proteins^35–37^. In our study, the expression of Akt was not significantly different among the different treatments. However, p-Akt expression was significantly increased in DN rats. Intriguingly, significant decreases in p-Akt were observed in the GSJD-treated groups, which were accompanied by decreased apoptosis. These data are in agreement with reports about the inhibitory effects of emodin^38^, berberine^39^ and astragaloside IV^40,41^, the bioactive constituents purified from the six herbs of GSJD, on p-Akt. Thus, our study suggested that the regulation of the mitochondrial apoptotic pathway and Akt pathway was most likely the basis for GSJD improving renal function in DN (Fig. 7). Although apoptosis plays a vital role in the progression of podocyte injury, there was no direct correlation between the expression of the BCL-2 family and Akt and podocytes in the present study. An *in vitro* cell-based assay system should be performed in further studies.

As mentioned in the Introduction, GSJD is a combination of six herbal extracts. We identified six compounds in GSJD by high-performance liquid chromatography (HPLC). However, it is difficult to determine all the active compounds in GSJD, which is a fundamental difference between Western and Chinese medicine. In addition, because the active compounds are not clear, the molecular mechanism of GSJD is not able to be researched in depth. Fortunately, some of the compounds purified from the six herbs of GSJD have been used to treat DN and gained satisfactory results. For instance, astragaloside IV^26,40–46^, berberine^39,47–51^, emodin^38,52^ and rhein^53,54^ could ameliorate DN by modulating multiple signalling pathways. Therefore, further investigation of the active compounds in GSJD is worthwhile. At the same time, we should recognize that systematic pharmacodynamics study is one of the most important developmental tendencies of complicated TCM.

In conclusion, this study provides evidence that GSJD attenuates albuminuria, Scr and BUN and prevents the progression of DN, subsequently reducing diabetes-induced apoptosis by a potential mechanism involving regulation of the mitochondrial apoptotic and Akt pathways. This study provided evidence of the beneficial effects of GSJD on improving DN development, which may result in an effective therapeutic medication for DN.

## Methods

### Preparation and identification of GSJD

All the dry raw herbs required for the preparation of GSJD were purchased from Beijing Guancheng Pharmaceutical Co., Ltd. (Beijing, China), and their identities were authenticated in accordance with the Chinese Pharmacopoeia (2015 Edition) by Professor Dan Yan (Beijing Shijitan Hospital, Capital Medical University of China). The recognized herbariums were stored in the Department of Pharmacy, Beijing Shijitan Hospital, Capital Medical University.

Semen *Euryales* (voucher specimen No. 20160301) and Fructus *Rosa laevigata* (voucher specimen No. 20160302) were extracted with boiling water (1:8) twice (2 h each time) and filtered. After standing for 12 h, the supernatant was concentrated to a relative density of 1.05~1.07 (65 °C) (extraction 1). In addition, Rhizome *Coptis chinensis* (voucher specimen No. 20160303), Rhizome *Rheum tanguticum* (voucher specimen No. 20160304), Radix *Astragalus membranaceus* (voucher specimen No. 20160305) and Radix *Angelica sinensis* (voucher specimen No. 20160306) were extracted with boiling water (1:8) twice (2 h each time) and filtered. The filtrates were concentrated to a relative density of 1.14~1.16 (60 °C). Alcohol was added to reach 70% (alcohol content). The alcohol-precipitation extracts were subsequently concentrated in a rotary evaporator under pressure to a relative density of 1.05~1.07 (65 °C) (extraction 2). Extractions 1 and 2 were lyophilized to form freeze-dried powder. Microcrystalline was added to the extracted powder as a filler, and 80% ethanol was used as a wetter. The compound was passed through a 120-mesh sieve and finally encased in a capsule in the proper proportion (Supplementary Fig. S3). The prepared GSJD capsules were stored at 4 °C, and the capsule content was diluted to the needed concentrations with 0.5% sodium carboxymethylcellulose before use.

To ensure the reliability and stability of the pharmacology experiment, HPLC was used to identify the major compounds and different batches of GSJD. As shown in Supplementary Fig. S4a–c, the representative active compounds alycosin-7-O-glucoside, berberine, rhein, emodin, chrysophanol and physcion were identified from the mixture standard. In addition, the high similarity of ten batches of GSJD achieved the requirement of stability of samples and ensured the reliability of pharmacodynamics (Supplementary Fig. S4d).

### Animals and treatment protocols

The animal experiment was conducted according to international, national and institutional rules regarding animal experimentation, and all procedures were approved by the Animal Ethics Committee, Beijing Shijitan Hospital, Capital Medical University, China (No. 2017-keyanlunshen-17).

Male Wistar rats (150 ± 20 g, 5 weeks) were obtained from Beijing Vital River Laboratory Animal Technology Co., Ltd. (Beijing, China; Certificate No. SCXK2016-0011). Rats were housed in a specific pathogen-free (SPF) environment within a temperature-controlled facility (21 ± 2 °C, 50 ± 5% relative humidity) under a 12 h light/dark cycle. Animals were allowed free access to tap water and standard chow for 1 week prior to the experiments.

The rats were randomly divided into the normal group (*n* = 6) and model group (*n* = 40) and provided *ad libitum* access to standard rodent chow and a HFD, respectively. The model was established by a HFD comprising standard chow supplemented with 10% lard, 20% sucrose, 2.5% cholesterol and 0.5% sodium cholate, obtained from Beijing Keao Xieli Feed Technology Co., Ltd. (Beijing, China). After 8 weeks of dietary manipulation, HFD-fed rats were injected intraperitoneally with 30 mg/kg STZ (Dalian Meilun Biotechnology, Dalian, China) diluted in citrate buffer (0.1 mol/L, pH 4.2)^27^. Three days after STZ injection, rats with blood glucose levels over 11.1 mmol/L^55^ were divided into the DN group (*n* = 6), fosinopril group (*n* = 6), GSJD low-dosage group (GSJD-L, *n* = 6), GSJD medium-dosage group (GSJD-M, *n* = 6) and GSJD high-dosage group (GSJD-H, *n* = 6). In clinic, GSJD at a dose of 4.05 g/d is used to treat patients weighing 60 kg, which is equivalent to 0.12 g/kg/d in the rats. Given this information, the dose of 0.12 g/kg/d was set as the low dose, 0.24 g/kg/d was set as the middle dose, and 0.48 g/kg/d was identified as the high dose. In addition, fosinopril was used as a positive control, and an equivalent dose of 1.05 mg/kg/day according to the human clinical dose was used for rats. The remaining 10 rats with blood glucose levels below 11.1 mmol/L were sacrificed. The normal control group rats (NC group) were injected with citrate buffer (0.1 mol/L, pH 4.2). Then, the model rats were left for 4 weeks to establish the DN model and allowed to continue feeding on their respective diets until the end of the study^56^.

GSJD suspensions in the different groups and fosinopril suspensions were given to rats by gastric gavage once a day for 8 weeks, while rats in the NC group and DN group were treated with 10 mL/kg distilled water. Eight weeks after administration, all rats were injected intraperitoneally with 10% chloral hydrate and sacrificed by cardiac puncture. Blood samples and kidneys were collected for the detection of various indicators. The experimental protocol (Supplementary Fig. S5) and dosage given to each group are shown in the supplementary materials.

### Measurement of general status and biochemical parameters

Body weight was measured every week. In the meantime, fasting blood glucose (FBG) was measured at 4-week intervals and monitored with a Roche ACCU-CHEK Active Glucometer (Roche Diabetes Care GmbH, Mannheim, Germany). Rats were housed in individual metabolic cages every 4 weeks for 24 h urine collection. Urinary protein excretion was determined by using a urine protein test kit (Nanjing Jiancheng Bioengineering Institute, China).

At the end of the study, blood samples were collected from the abdominal aorta. Serum was separated (3,500 r/min, 10 min) for the detection of Scr, BUN, total cholesterol (TC), triglycerides (TG), total protein (TP) and serum albumin (ALB) by an automatic biochemistry analyser (Chemray 240, Rayto Life and Analytical Sciences Co., Ltd, China).

The right kidneys of rats in each group were removed and weighed after blood collection. The kidney hypertrophy index (KHI) was calculated according to the method described previously, that is, KHI = kidney weight (KW)/BW^57^.

### Histological examination of the kidneys

The left kidneys were removed and immediately cut into three segments. One segment (1 cm^3^) was fixed in 4% paraformaldehyde (PFA), dehydrated, embedded in paraffin and cut into 5 μm-thick sections. Renal sections were stained with haematoxylin-eosin (H&E), periodic acid-Schiff (PAS) and Masson’s trichrome to evaluate glomerulosclerosis, tubular injury and tubulointerstitial damage. The stained specimens were inspected by an independent blinded pathologist by utilizing a Nikon Eclipse E200 (Nikon Corporation, Tokyo, Japan) light microscope. Semiquantitative scoring with the glomerulosclerosis index (GSI) and tubular interstitial fibrosis index (TIFI) was performed in a blinded manner using a method described previously^58,59^. Forty glomeruli randomly selected from each rat were scored from six rats in each group. The remaining segments and the right kidneys were immediately frozen in liquid nitrogen and stored at −80 °C until assay.

### Transmission electron microscopy detection

From the renal issues, the other 1 mm^3^ segment of tissue was fixed with 2.5% glutaraldehyde for 24 h, washed with PBS, fixed with 1% osmium tetroxide, dehydrated by washing with a series of PBS, ethanol and acetone, and embedded with embedding medium. The glomerular basement membranes (GBMs) and podocytes were stained, and ultrastructural changes were observed with a HITACHI-HT7700 transmission electron microscope. GBM thickness was directly measured and calculated with Image-Pro Plus 6.0 software on the basis of the method described previously^58^.

### Western blot analysis

Total protein was extracted from rat renal tissues with RIPA lysis buffer (P1003B, Beyotime, China) and phenylmethanesulfonylfluoride (PMSF, ST506, Beyotime, China). After centrifugation of homogenate, supernatant was used for determination of protein concentration with a BCA protein assay reagent (P0012, Beyotime, China). Equal amounts of proteins were boiled in SDS-PAGE sample loading buffer (P0015, Beyotime, Beijing, China) for 10 min, separated by 10% sodium dodecyl sulfate-polyacrylamide gel electrophoresis and transferred to pure nitrocellulose blotting membranes (66485, Pall Life Sciences, USA). The membranes were blocked with 5% skim milk in TBST (20 mM Tris-Cl, 150 mM NaCl, 0.05% (v/v) Tween-20, pH 7.4) for 1 h at room temperature. Subsequently, membranes were incubated with primary antibodies overnight at 4 °C, followed by incubation with Alexa Fluor 790 goat anti-rabbit IgG H&L (ab186697, Abcam, Cambridge, UK) or Alexa Fluor 680 goat anti-mouse IgG (ab186694, Abcam, Cambridge, UK) secondary antibodies at a dilution of 1:10000 for 1 h at room temperature. Finally, fluorescent signals were collected using an Odyssey infrared imaging system (LI-COR, Lincoln, NE, USA). Primary antibodies used for detection of Akt (1:1000, #4691) and p-Akt (1:2000, Ser473, #4060) were purchased from Cell Signaling Technology (Danvers, USA), and those for detection of Bax (1:800, ab32503), Bcl-2 (1:1000, ab59348), nephrin (1:5000, ab136894) and podocin (1:1000, ab50339) were purchased from Abcam (Cambridge, UK). Band intensities were quantified using ImageJ software and normalized to the respective controls.

### TUNEL staining

To detect DNA fragmentation in the cell nuclei (a marker for apoptosis in renal tissues), a TUNEL assay was performed using an *in situ* cell death detection kit (Roche Applied Science, Penzberg, Upper Bavaria, Germany) in accordance with the manufacturer’s instructions. DAPI was used to label the nuclei. Localized green fluorescence of apoptotic cells was detected against a blue background using fluorescence microscopy. TUNEL-positive cells were directly calculated with ImageJ software on the basis of the method described previously^59^.

### Statistical analysis

Data are presented as the mean ± standard error of the mean (SEM) unless stated otherwise. The data were analysed using GraphPad Prism version 6.01 (GraphPad Software Inc, La Jolla, CA, USA). One-way analysis of variance (ANOVA) with a post hoc Student-Newman-Keuls test and unpaired two-tailed Student’s *t*-test were used to assign significance. Differences between groups with *P* < 0.05 and *P* < 0.01 were considered statistically significant.

## Supplementary information

Supplementary Information.

## Data Availability

All data generated during this study are included in this published article and the raw data are available from the corresponding author on reasonable request.
