# Supplementary material for: Renoprotective effects of Gushen Jiedu capsule on diabetic nephropathy in rats
Source: Sci Rep. 2020 Feb 6;10:2040. doi: 10.1038/s41598-020-58781-2 (PMC7005167; doi:10.1038/s41598-020-58781-2)
Supplement: Supplementary file 1 — Supplementary Information. [file 41598_2020_58781_MOESM1_ESM.pdf]

Supplementary Information

Renoprotective effects of Gushen Jiedu capsule on diabetic nephropathy in rats

Lei Zhang[1,2,†], Zhirui Yang[1,2,†], Yidan Zhao[1,2], Xinyu Yang[1,2], Xintong Meng[1,2], Juan Liu[1,2], Yi Liu[1,2], Can Yan[3,*], and Dan Yan[1,2,*]

[1]Beijing Shijitan Hospital, Capital Medical University, Beijing 100038, China

[2]Beijing Key Laboratory of Bio-characteristic Profiling for Evaluation of Rational Drug Use, Beijing 100038, China

[3]Guangzhou University of Chinese Medicine, School of Basic Medical Sciences, Guangzhou 510006, China

[*]Corresponding author

Can Yan

E-mail: yanc020@126.com

Dan Yan

E-mail: pharmsci@126.com

[†]These authors contributed equally to this work.

As shown in Supplementary Fig. S1, Gushen Jiedu capsule and the six herbal components in GSJD were photographed by Lei Zhang. In addition, the ancient book (Hong Shi Ji Yan Fang) is a published book and is one of the classical textbooks of TCM. It was compiled by Zun Hong and first recorded in the Song Dynasty (1170 AD) in China. Lei Zhang borrowed it from the Capital Medical University Library and photographed it for Supplementary Fig. S1.

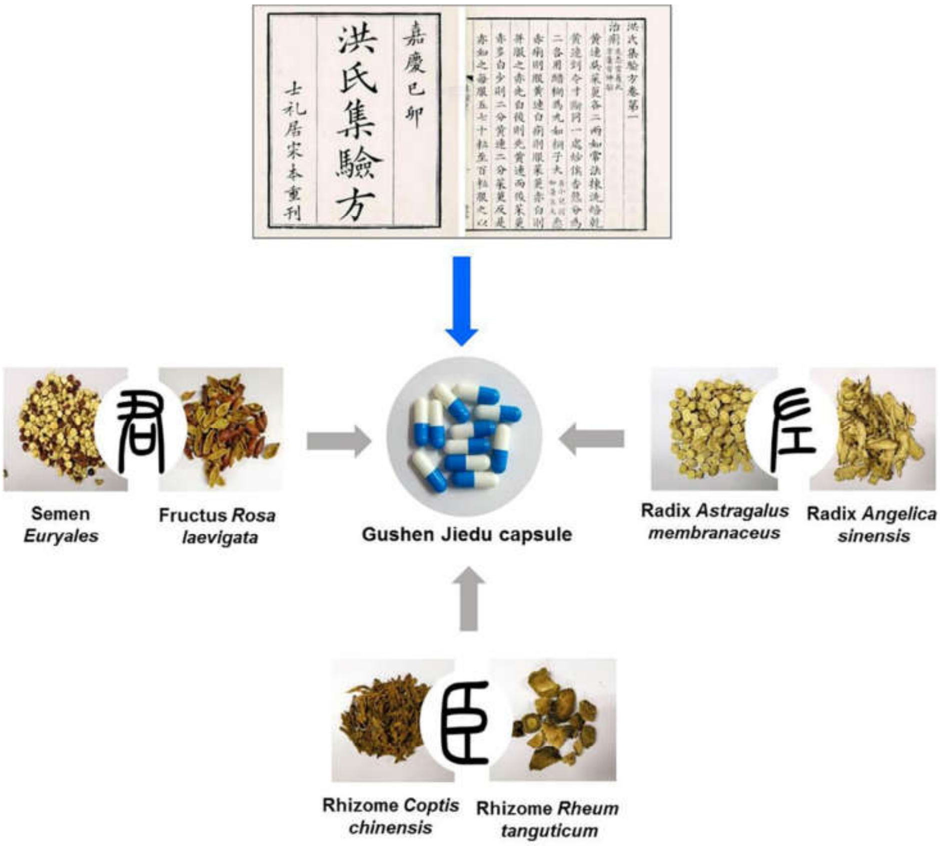

Supplementary Figure S1. Stages in the medicalization of Gushen Jiedu formula. It was recorded that this formula was cultivated from "Shuilu Erxian Dan" in the Song Dynasty (1170 AD) in China and was used to treat early-stage DN. Gushen Jiedu capsule (GSJD) contains the following six herbs: Semen *Euryales*, Fructus *Rosa laevigata*, Rhizome *Coptis chinensis,* Rhizome *Rheum tanguticum,* Radix *Astragalus membranaceus* and Radix *Angelica sinensis.* In

the formula, Semen *Euryales* and Fructus *Rosa laevigata* are the monarch components, and Rhizome *Coptis chinensis* and Rhizome *Rheum tanguticum* are the minister components, while Radix *Astragalus membranaceus* and Radix *Angelica sinensis* are the adjuvant components.

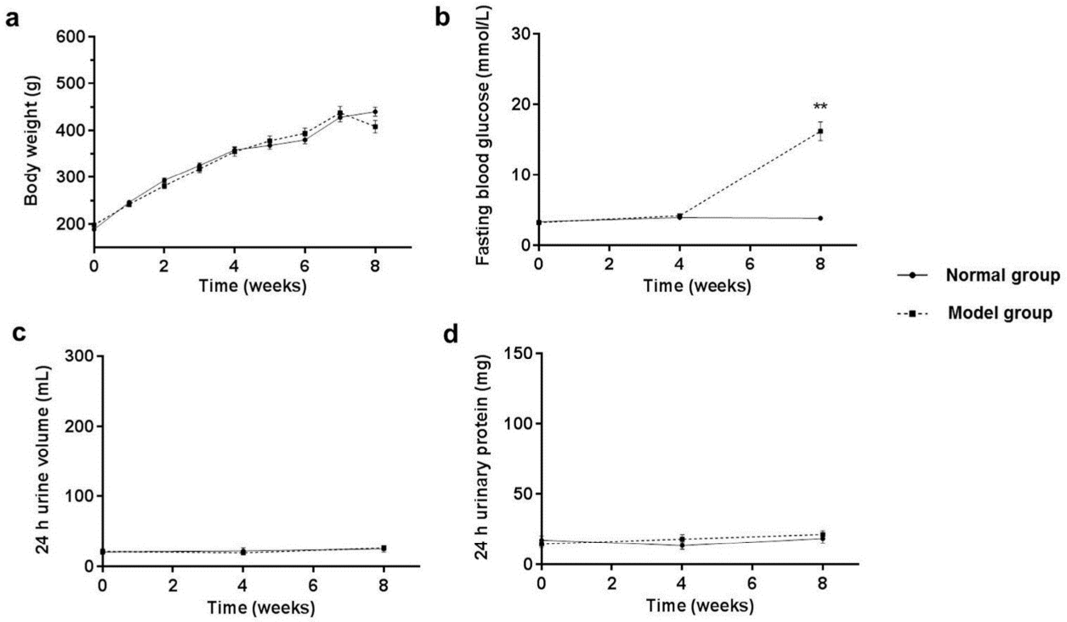

Supplementary Figure S2. The (**a**) body weight, (**b**) fasting blood glucose, (**c**) 24 h urine volume and (**d**) 24 h urinary protein of the HFD-induced diabetic rat model. Data were analysed by *t*-tests and presented as the mean ± SEM ($n = 6$). $^{**}P < 0.01$ *vs*. normal group.

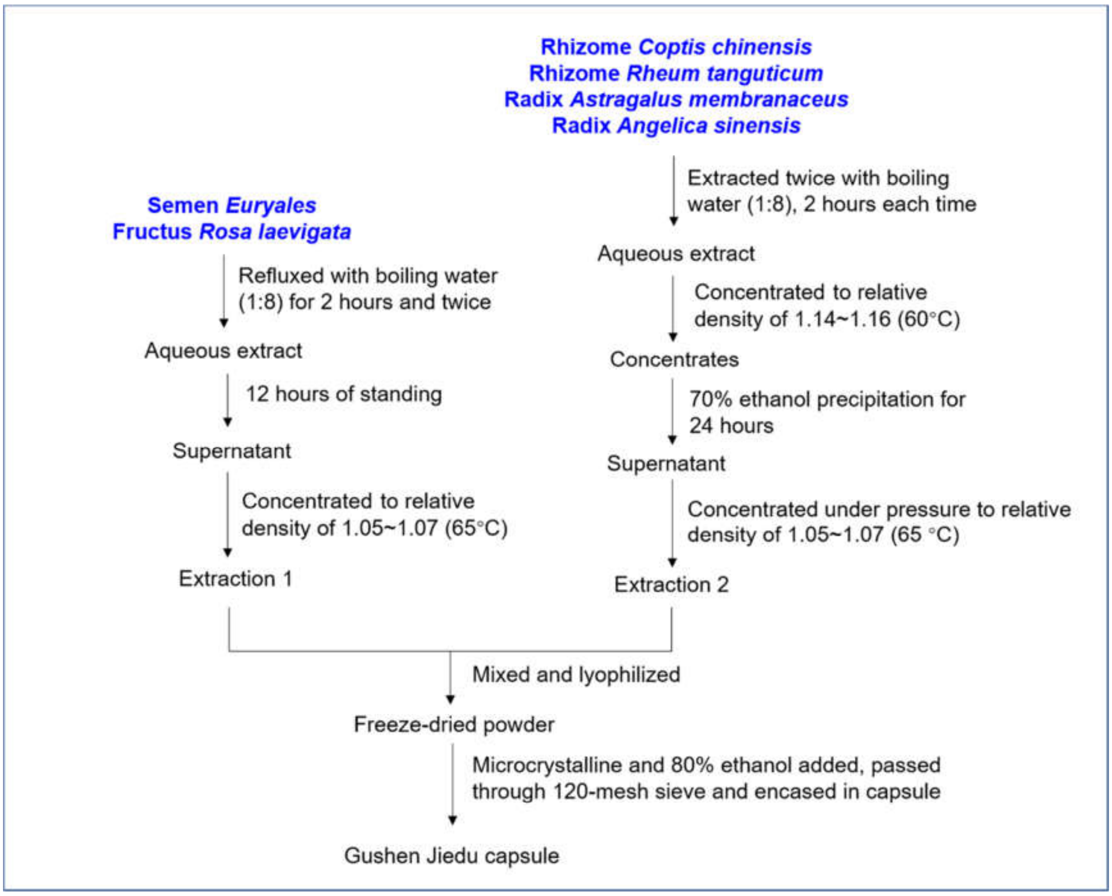

Supplementary Figure S3. The extraction and preparation process of GSJD.

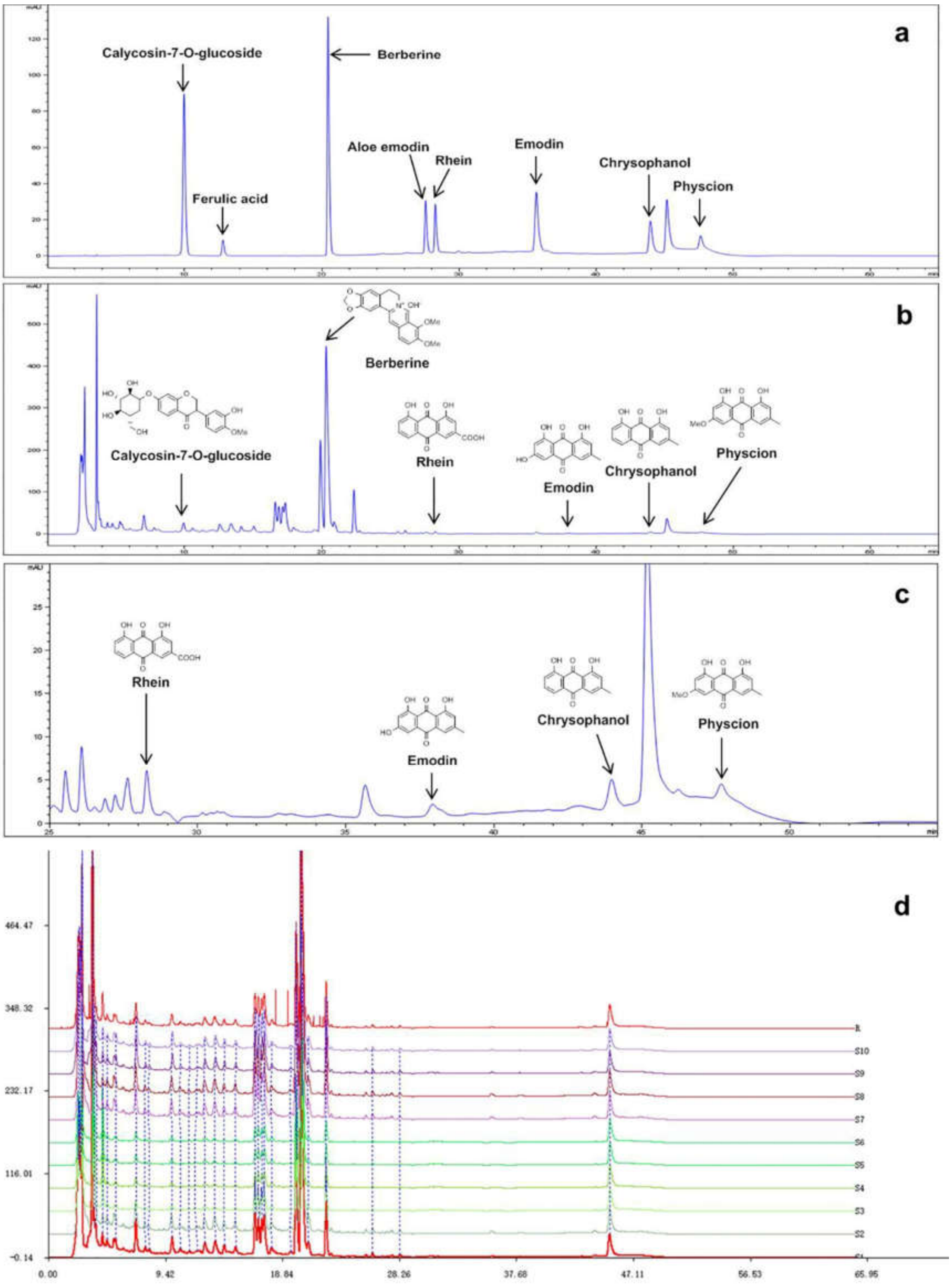

Supplementary Figure S4. HPLC chromatograms of (**a**) mixed standards, (**b**) samples of GSJD, (**c**) partial enlarged detail of GSJD and (**d**) the fingerprint of ten batches of GSJD.

HPLC conditions

Sample and standard preparation

Before use, the GSJD was stored in an exsiccator and protected from light. GSJD powder (0.850 g) was extracted with 50.0 mL of MeOH for 1.0 h. After filtering, the filtrate was concentrated to 10.0 mL. The standard solutions (in methanol) of calycosin-7-O-glucoside, ferulic acid, berberine, aloe emodin, rhein, emodin, chrysophanol and physcion were prepared at concentrations of 513.0, 696.0, 909.0, 421.0, 360.0, 394.0, 408.0 and 445.0 μg/mL, respectively. A mixture of standard stock solutions containing the above six compounds was prepared and serially diluted to concentrations of 51.3, 12.5, 90.9, 16.8, 15.8, 15.8, 16.3 and 8.0 μg/mL.

Chromatographic conditions, detection and integration settings

Chromatography experiments were performed on an Agilent 1260 HPLC system with a Phenomenex Luna C_{18} column (4.6 ×250 mm, 5 μm, Phenomenex Corporation, USA). Separation was performed at a flow rate of 1.0 mL/min by gradient conditions (0.2% aqueous phosphoric acid (containing 0.03 mol/L sodium dihydrogen phosphate) as phase A and acetonitrile-water 10:1 (v/v, containing 0.24% phosphoric acid) as phase B: 0–5 min, 80–78% A; 5–8 min, 78–75% A; 8–12 min, 75–70% A; 12–15 min, 70–65% A; 15–20 min, 65–50% A; 20–25 min, 50–40% A; 25–35 min, 40–35% A; 35–40 min, 35–25% A; 40–45 min, 25–20% A; 45–50 min, 20–80% A; 50–65 min, 80% A). The sample injected into the system for analysis was 10 μL. The oven temperature was 35°C with detection at 280 nm.

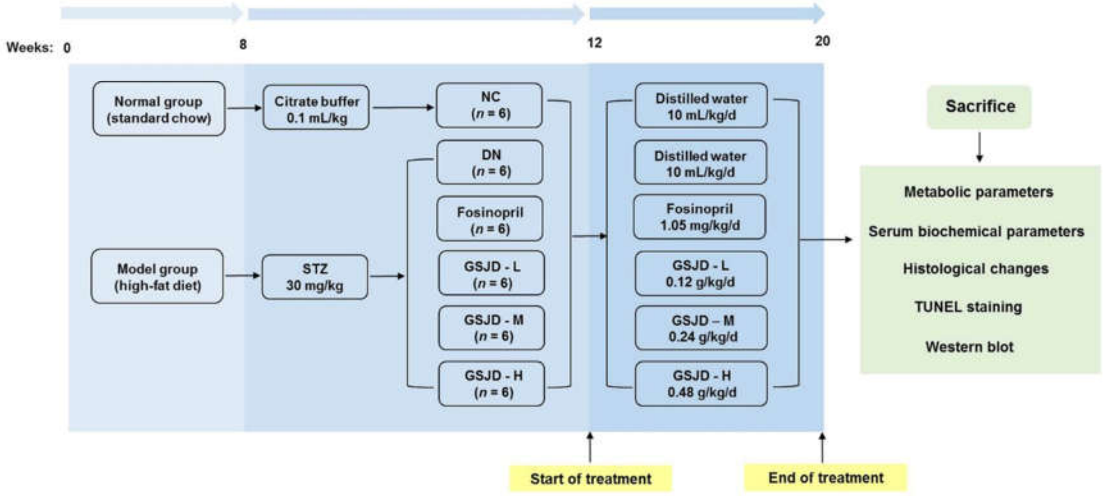

Supplementary Figure S5. Schematic representation of the experimental protocol.

Animals were allowed free access to tap water and standard chow for 1 week prior to experiments. At the start of the experiment, the rats were randomly divided into a normal group (n = 6) and a model group (n = 40). The diabetic nephropathy model was established by HFD comprising standard chow supplemented with 10% lard, 20% sucrose, 2.5% cholesterol and 0.5% sodium cholate. After 8 weeks of dietary manipulation, HFD-fed rats were injected intraperitoneally with a low dose of STZ (30 mg/kg, Dalian Meilun Biotechnology, China) diluted in citrate buffer (0.1 mol/L, pH 4.2). Three days after STZ injection, rats with blood glucose levels over 11.1 mmol/L were divided into the DN group (n = 6), fosinopril group (n = 6), GSJD low dosage group (GSJD-L, n = 6), GSJD medium dosage group (GSJD-M, n = 6) and GSJD high dosage group (GSJD-H, n = 6). The remaining 10 rats with blood glucose levels below 11.1 mmol/L were sacrificed. The normal control group rats (NC group) were injected with citrate buffer (0.1 mol/L, pH 4.2). Then, the model rats were left for 4 weeks to establish the DN model and allowed to continue feeding on their respective diets until the end of the study.

The dosage given to each group was generated for the current study: (1) normal control rats treated with distilled water (*n* = 6 and presented as NC), (2) diabetic rats treated with distilled water (*n* = 6 and presented as DN), (3) diabetic rats treated with fosinopril (1.05 mg/kg/d, *n* = 6 and presented as fosinopril), (4) diabetic rats treated with low-dose GSJD (0.12 g/kg/d, *n* = 6 and presented as GSJD-L), (5) diabetic rats treated with medium-dose GSJD (0.24 g/kg/d, *n* = 6 and presented as GSJD-M), and (6) diabetic rats treated with high-dose GSJD (0.48 g/kg/d, *n* = 6 and presented as GSJD-H).

At the end of the study, all rats were sacrificed and examined by serum biochemical, urinary, histological, TUNEL and western blot analysis.

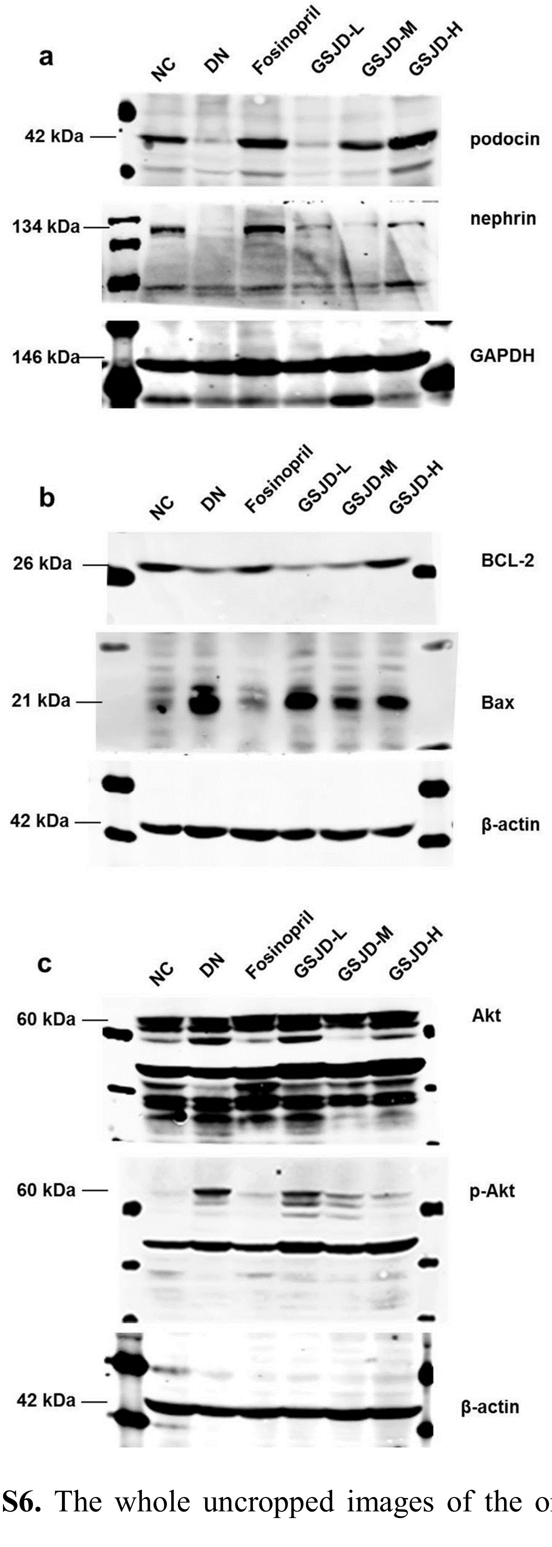

Supplementary Figure S6. The whole uncropped images of the original western blots of (**a**) podocin, (**a**) nephrin, (**b**) BCL-2, (**b**) Bax, (**c**) Akt and (**c**) p-Akt.
